# Isolation and functional characterization of *JcFT*, a *FLOWERING LOCUS T* (*FT*) homologous gene from the biofuel plant *Jatropha curcas*

**DOI:** 10.1186/1471-2229-14-125

**Published:** 2014-05-08

**Authors:** Chaoqiong Li, Li Luo, Qiantang Fu, Longjian Niu, Zeng-Fu Xu

**Affiliations:** 1 Key Laboratory of Tropical Plant Resources and Sustainable Use, Xishuangbanna Tropical Botanical Garden, Chinese Academy of Sciences, Menglun, Yunnan 666303, China; 2 University of Chinese Academy of Sciences, Beijing 100049, China; 3 Key Laboratory of Gene Engineering of the Ministry of Education, and State Key Laboratory for Biocontrol, School of Life Sciences, Sun Yat-sen University, Guangzhou, Guangdong 510275, China; 4 School of Life Sciences, University of Science and Technology of China, Hefei, Anhui 230027, China

**Keywords:** Biofuel, Early flowering, Florigen, FLOWERING LOCUS T, Physic nut

## Abstract

**Background:**

Physic nut (*Jatropha curcas* L.) is a potential feedstock for biofuel production because *Jatropha* oil is highly suitable for the production of the biodiesel and bio-jet fuels. However, *Jatropha* exhibits low seed yield as a result of unreliable and poor flowering. *FLOWERING LOCUS T* (*FT*) –like genes are important flowering regulators in higher plants. To date, the flowering genes in *Jatropha* have not yet been identified or characterized.

**Results:**

To better understand the genetic control of flowering in *Jatropha*, an *FT* homolog was isolated from *Jatropha* and designated as *JcFT*. Sequence analysis and phylogenetic relationship of *JcFT* revealed a high sequence similarity with the *FT* genes of *Litchi chinensis*, *Populus nigra* and other perennial plants. *JcFT* was expressed in all tissues of adult plants except young leaves, with the highest expression level in female flowers. Overexpression of *JcFT* in *Arabidopsis* and *Jatropha* using the constitutive promoter cauliflower mosaic virus 35S or the phloem-specific promoter *Arabidopsis* SUCROSE TRANSPORTER 2 promoter resulted in an extremely early flowering phenotype. Furthermore, several flowering genes downstream of *JcFT* were up-regulated in the *JcFT*-overexpression transgenic plant lines.

**Conclusions:**

*JcFT* may encode a florigen that acts as a key regulator in flowering pathway. This study is the first to functionally characterize a flowering gene, namely, *JcFT*, in the biofuel plant *Jatropha*.

## Background

Physic nut (*Jatropha curcas* L.) is a perennial plant that belongs to the Euphorbiaceae family, and is monoecious with male and female flowers borne on the same plant within the same inflorescence [1]. The potential benefit of growing *Jatropha* as a cash crop for biofuel in tropical and sub-tropical countries is now widely recognized [2-4]. *Jatropha* has been propagated as a unique and potential biodiesel plant owing to its multipurpose value, high oil content, adaptability to marginal lands in a variety of agro-climatic conditions, non-competitiveness with food production, and high biomass productivity [2,5]. The oil content of *Jatropha* seeds and the kernels ranges from 30% to 50% and 45% to 60% by weight, respectively. Oil from *Jatropha* contains high levels of polyunsaturated fatty acids, and it is therefore suitable as a fuel oil [6,7]. However, the potential of *Jatropha* as a biofuel plant is limited by its low seed production. Despite the clear evidence of the abundant biomass generated by *Jatropha*, it is not indicative of high seed productivity [8]. There are too many vegetative shoots in *Jatropha*, which could develop into reproductive shoots under suitable conditions. It is therefore imperative to reduce undesired vegetative growth. In addition to these considerations, unreliable and poor flowering are important factors that contribute to low seed productivity in *Jatropha *[9]. The *FLOWERING LOCUS T* (*FT*) gene plays a crucial role in the transition from vegetative growth to flowering, which is a potent factor integrating the flowering signals. In this context, the function of *JcFT*, an *FT* homolog in *Jatropha*, was analyzed to improve the understanding of the flowering mechanism in *Jatropha*, which will be critical for the genetic improvement of this species.

The transition from vegetative to reproductive growth in plants is regulated by both environmental and endogenous cues [10]. The genetic network of flowering has been investigated primarily in the model plant *Arabidopsis*, and five major genetically pathways control flowering initiation: the photoperiod, vernalization, gibberellin, autonomous and age pathways [11]. Recent advances in transgenic plants and traditional grafting studies have revealed that FT protein acts as a mobile flowering signal, whose ability to induce flowering involves long-distance transport [12,13]. The findings of many studies have helped establish the role of FT as a floral pathway integrator that respond to both environmental and endogenous flowering signals [14].

In *Arabidopsis*, *FT* is expressed in leaf phloem, and the FT protein subsequently moves to the shoot apex, where it forms a complex with the basic domain/leucine zipper protein FD. This FT/FD heterodimer activates the downstream floral meristem identity gene *APETALA1* (*AP1*) [12,15,16]. *FT*-like genes have been isolated from many plants, including tomato [17], pumpkin [18], rice [19], barley [20], grape [21], apple [22], and potato [23], and the function of most *FT* genes is conserved [24].

In this study, we cloned and characterized the *Jatropha FT* homolog, *JcFT*. We also analyzed the function of *JcFT* in floral induction using transgenic *Arabidopsis* and *Jatropha*.

## Results

### Cloning and sequence analysis of *JcFT*

A combined reverse transcriptase-polymerase chain reaction (RT-PCR) and rapid-amplification of cDNA ends (RACE) strategy was used to isolate an *FT*-like cDNA from *Jatropha. JcFT* cDNA (GenBank accession no. KF113881) encoded a 176-amino acid protein with 89%, 83%, 80%, and 78% sequences identity with *Litchi chinensis* LcFT [25], *Citrus unshiu* CiFT [26], rice Hd3a [27], and *Arabidopsis* FT [28], respectively. The molecular weight and isoelectric point of the deduced protein were 20.03 kDa and 6.82, respectively.

**Figure 1:**
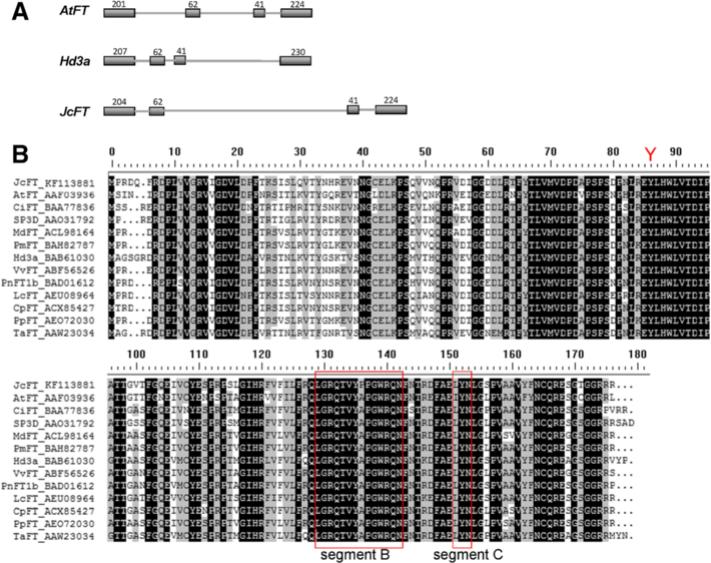
**Comparison of *JcFT *and other *FT*-like genes. (A)** Gene structures of *JcFT*, *Hd3a*, and *AtFT*. Boxes indicate exons and thin lines indicate introns. Exon sizes are indicated above each box. **(B)** Sequence alignment of amino acid sequences. Identical amino acid residues are shaded in black, and similar residues are shaded in gray. Dots denote gaps. Boxes indicating the 14-amino-acid stretch (segment B) and the LYN triad (segment C), and "Y" indicating the highly conserved amino acid Tyr (Y).

The genomic sequence of *JcFT* consisted of four exons, which resembles the genomic structure of other *FT* genes (Figure 1A). A multiple alignment was performed using the JcFT sequence and the sequences of FT homologs from other species (Figure 1B). The conserved key amino acid residue Tyr (Y) found in FT homologs was identified at position 85 of the JcFT protein (Figure 1B). JcFT also contained two highly similar sequences to *Arabidopsis* FT in the 14-AA stretch known as “segment B” and in the LYN triad in “segment C” [29] (Figure 1B).

**Figure 2:**
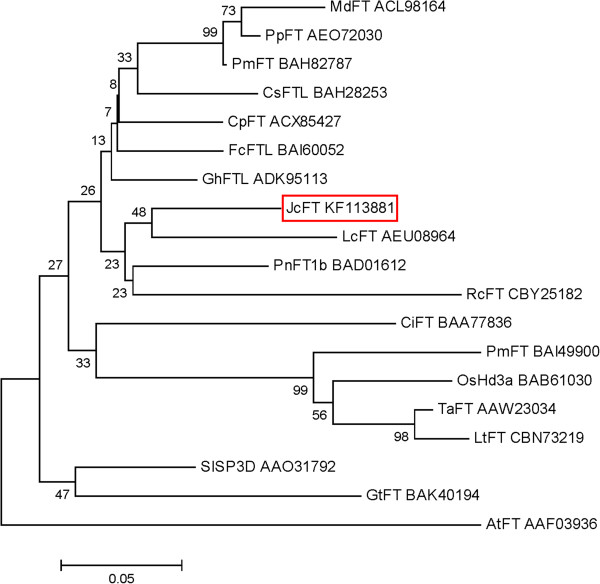
**Phylogenetic analysis of the FT homologs from different plant species.** Species abbreviations: At, *Arabidopsis thaliana*; Ci, *Citrus unshiu*; Cp, *Carica papaya*; Cs, *Cucumis sativus*; Fc, *Ficus carica*; Gh, *Gossypium hirsutum*; Gt, *Gentiana triflora*; Jc, *Jatropha curcus*; Lc, *Litchi chinensis*; Lt, *Lolium temulentum*; Md, *Malus domestica*; Os, *Oryza sativa*; Phm, *Phyllostachys meyeri*; Pm, *Prunus mume*; Pn, *Populus nigra*; Pp, *Prunus persica*; Rc, *Rosa chinensis*; Sl, *Solanum lycopersicum*; Ta, *Triticum aestivum*.

A phylogenetic tree was constructed to analyze the phylogenetic relationship between JcFT and the FTs from other angiosperms (Figure 2). The analysis revealed that the JcFT protein (indicated with a red-boxed) was more closely related to the FTs of perennial woody plants such as *Litchi chinensis*, instead of annual herbaceous plants such as *Arabidopsis*.

### Expression pattern of *JcFT* in *Jatropha*

**Figure 3:**
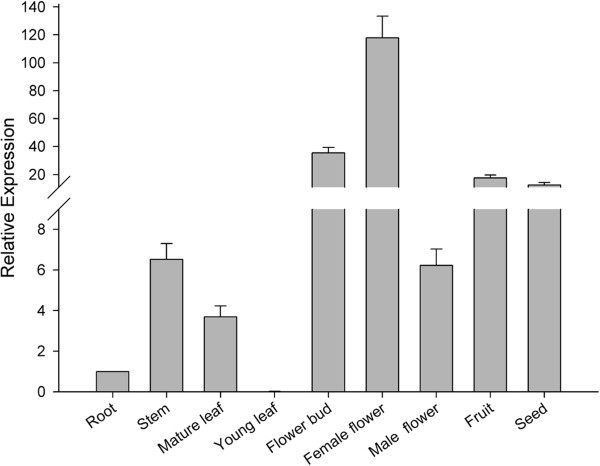
**Expression of *JcFT *in various organs of three-year-old adult *Jatropha*.** The qRT-PCR results were obtained from two independent biological replicates and three technical replicates for each sample. The levels of detected amplicons were normalized using the amplified products of the *JcActin1*. The mRNA level in the root tissue was set as the standard with a value of 1.

To assess the expression pattern of *JcFT* in *Jatropha*, we performed a quantitative RT-PCR (qRT-PCR) analysis using the specific primers listed in Table S1. *JcFT* was expressed in all adult plants tissues except young leaves (Figure 3). Interestingly, *JcFT* was primarily expressed in the reproductive organs rather than the leaves, where expression of a florigen-encoding gene is expressed (Figure 3).

### Constitutive overexpression and phloem-specific expression of *JcFT* in *Arabidopsis* induces early flowering and complements the *ft-10* mutant phenotype

**Figure 4:**
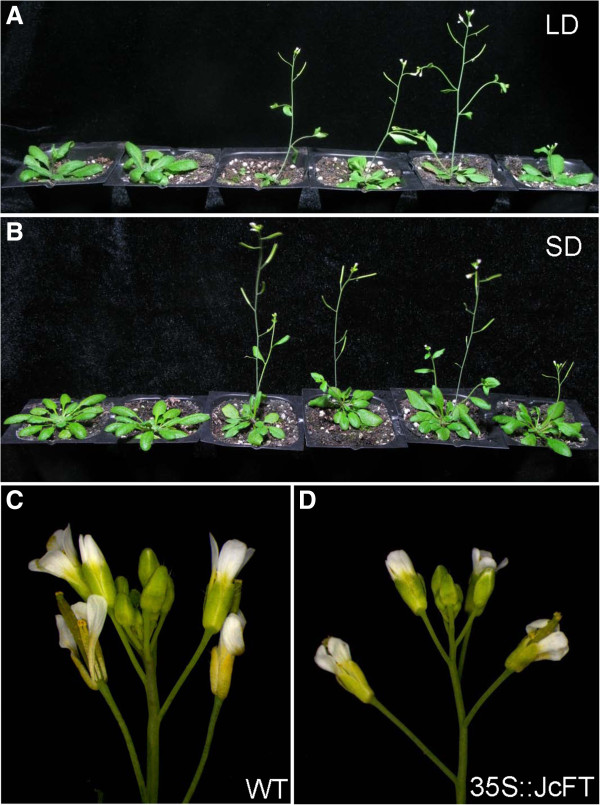
**Ectopic expression of *JcFT *causes early flowering in transgenic *Arabidopsis.*** Growth under LD conditions **(A)** and SD conditions **(B)** at 28 days and 45 days after germination, respectively. Left to right: WT, *ft-10*, 35S::*JcFT* in Col, SUC2::*JcFT* in Col, 35S::*JcFT* in *ft-10*, and SUC2::*JcFT* in *ft-10*. (**C** and **D**) Inflorescences of WT and 35S::*JcFT* transgenic plants.

**Figure 5:**
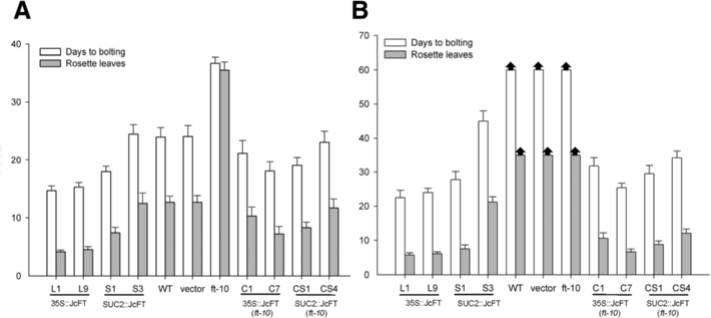
**Ectopic expression of *JcFT *affects flowering in *Arabidopsis*. (A)** Days and leaves to bolting for several *JcFT* overexpression (CaMV 35S) and phloem- specific expression (SUC2) transgenic *Arabidopsis* lines, empty vector-transformed plants, WT and mutant *ft-10* plants grown under LD conditions. **(B)** Days and leaves to bolting for transgenic lines in the Col and *ft-10* background grown under SD conditions. Values are means ± SD of the results from ten plants of each transgenic line. Arrows at the top of bars for WT, empty vector-transformed Col and *ft-10* indicate that plants have not flowered.

To determine whether *JcFT* is involved in the regulation of flowering time, *JcFT* cDNA driven by the constitutive cauliflower mosaic virus 35S (CaMV 35S) promoter or the phloem-specific *Arabidopsis* SUCROSE TRANSPORTER 2 (SUC2) promoter was transformed into wild-type *Arabidopsis* Columbia (WT) and *ft-10* mutant plants. An empty vector was transformed into WT as a control. Transgenic plants were confirmed by RT-PCR analysis of *JcFT* expression (Additional file 1: Figure S1A). Twenty-four and seven independent T_0_ transgenic lines were generated with the 35S::*JcFT* construct in WT and *ft-10* mutant, respectively. For most of these lines, bolting occurred significantly earlier than in WT and *ft-10* plants under inductive long-day (LD) conditions (Figures 4A and 5A).

We selected four independent homozygous lines in the T_2_ generation to examine the phenotypes. The L1 and L9 lines were created by transforming WT with the 35S::*JcFT* construct, and the C1 and C7 lines harbored the construct in the *ft-10* mutant background. Lines L1 and L9 bolted 8–14 days earlier and produced 6–11 fewer rosette leaves than the WT control under LD conditions, whereas no differences in bolting time were observed when comparing WT and the transgenic lines transformed with the empty vector (Figure 5A). Under non-inductive short-day (SD) conditions, all transgenic plants flowered much earlier than WT and the *ft-10* mutant, both of which did not flower until 60 days after sowing in soil (Figures 4B and 5B). *JcFT* overexpression in *Arabidopsis* did not cause any defects in flower development (Figure 4C and 4D), but it did significantly reduce vegetative growth time. Further analysis indicated that the promotion of flowering in 35S::*JcFT* transgenic *Arabidopsis* was correlated with a significant up-regulation of the flower meristem identity genes *AP1* and *LEAFY* (*LFY*) (Additional file 2: Figure S1).

To determine whether *FT*-like genes are functionally conserved and active in vascular tissue, the phloem-specific promoter SUC2 has been used to drive the expression of *FT*-like genes in *Arabidopsis* and other species [12,30-32]. We obtained ten WT and eight *ft-10* independent T_0_ transgenic lines harboring the SUC2::*JcFT* construct. The S1 and S3 lines were created by transforming WT with the SUC2::*JcFT* construct, and the CS1 and CS4 lines harbored the construct in the *ft-10* mutant background. Similar to the observations for the 35S::*JcFT* transgenic lines, lines S1 and CS1 flowered much earlier than WT and *ft-10*, respectively. Lines S3 and CS4 flowered at approximately the same time and produced as many leaves as WT (24 days, 12 leaves) or flowered slightly earlier (Figure 5A) under LD conditions. Similar to the 35S::*JcFT* transgenic lines, all the SUC2::*JcFT* transgenic lines flowered earlier than WT and *ft-10* under SD conditions (Figure 5B).

Taken together, these findings demonstrated that ectopic expression of *JcFT* in *Arabidopsis* resulted in an early flowering phenotype.

### Overexpression of *JcFT* in *Jatropha* causes early flowering *in vitro*

**Figure 6:**
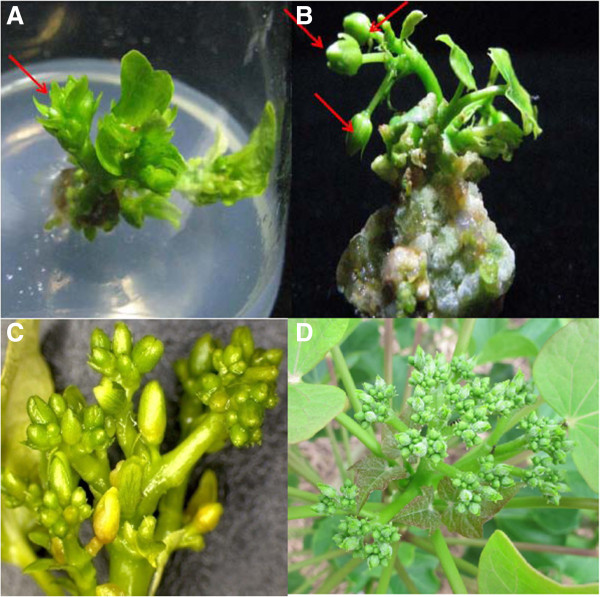
**Early flowering of 35S::*JcFT *transgenic *Jatropha *cultured *in vitro. *(A and B)** Flower buds of transgenic *Jatropha* cultured *in vitro* for seven weeks. **(C)** Inflorescence of transgenic *Jatropha* cultured *in vitro*. **(D)** Inflorescence of wild *Jatropha* in the field. Red arrows indicate flower buds.

The transgenic analysis in *Arabidopsis* suggested that *JcFT* could be a floral activator in *Jatropha*. To test whether *JcFT* similarly resulted in an early-flowering phenotype in *Jatropha*, we generated transgenic *Jatropha* with the 35S::*JcFT* construct used for *Arabidopsis* transformation. Mature *Jatropha* cotyledons were used as explants for transformation, as previously described [33]. To our surprise, flower buds initiated directly from the *Agrobacterium*-transformed calli after *in vitro* culture for seven weeks (Figure 6A and 6B), whereas the control explants never produced flower buds under the same conditions. The *in vitro* cultured transgenic *Jatropha* also produced intact inflorescences, but the inflorescences did not produce as many small flowers as wild *Jatropha* in the field (Figure 6C and 6D). Nevertheless, these findings demonstrate that JcFT is a powerful inducer of flowering in *Jatropha*.

**Figure 7:**
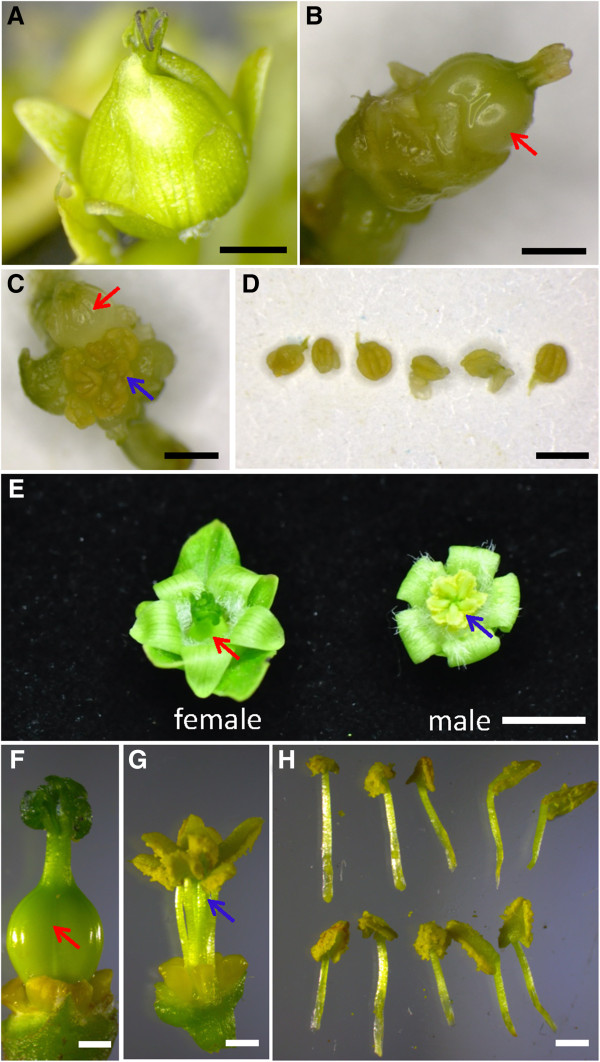
**Abnormal flowers of transgenic *Jatropha *harboring 35S::*JcFT*. (A)** A female flower of transgenic *Jatropha* cultured *in vitro*. **(B)** Pistil of a transgenic female flower. **(C)** An abnormal hermaphrodite flower of transgenic *Jatropha* cultured *in vitro*. **(D)** Abnormal stamens from an abnormal hermaphrodite flower of transgenic Jatropha shown in **(C). (E)** Normal female and male flowers of wild *Jatropha* grown in the field. **(F)** Pistil of a wild-type female flower. (**G** and **H**) Stamens of a wild-type male flower. Bars in **(A)**-**(D)** and **(F)**-**(H)** represent 1 mm, and bar in **(E)** represents 5 mm. Red arrows indicate pistils, and blue arrows indicate stamens.

Although flower buds were produced *in vitro*, most were abortive and wilted several weeks later. A few flower buds developed into flowers (Figure 7A and 7C), but these flowers also wilted. Furthermore, these *in vitro* flowers were abnormal; for example, the petals of the female flower could not spread (Figure 7A). By removing the sepals and petals of female flower, the pistil was made visible (Figure 7B). Compared with the wild-type female flower (Figure 7F), the stigma of transgenic female flower was shorter (Figure 7B). An abnormal *in vitro* hermaphrodite flower of transgenic *Jatropha* (Figure 7C) had six stamens with very short filaments (Figure 7D) in contrast to the normal male flower (Figure 7E, G), which has ten stamens (Figure 7H). Consequently, no regenerated transgenic plants harboring 35S::*JcFT* were obtained.

**Figure 8:**
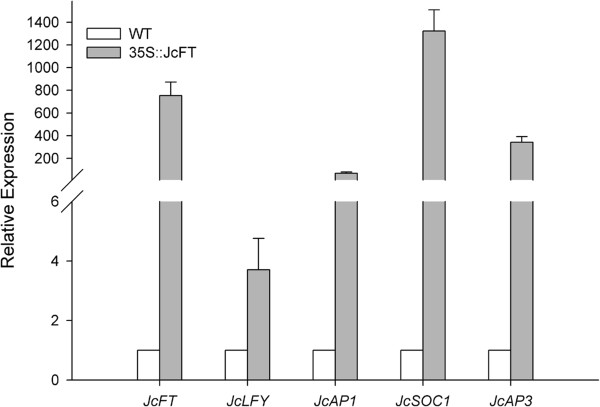
**Quantitative RT-PCR analysis of flowering genes downstream of *JcFT *in WT and 35S::*JcFT *transgenic *Jatropha*.** The qRT-PCR results were obtained using two independent biological replicates and three technical replicates for each RNA sample extracted from apex of the *35S::JcFT* transgenic and wild-type (WT) shoots cultured *in vitro*. Transcript levels were normalized using *JcActin1* gene as a reference. The mRNA level in WT was set as the standard with a value of 1.

To determine whether *JcFT* overexpression in the transgenic *in vitro* flowering lines altered the expression of downstream flowering genes, such as *SUPPRESSOR OF OVEREXPRESSION OF CONSTANS 1* (*SOC1*), *LFY*, and *AP1* homologs in *Jatropha *[11], qRT-PCR analysis was performed with RNA extracted from apex of the *35S::JcFT* transgenic and wild-type shoots cultured *in vitro*. As expected, the transcript levels of *JcLFY*, *JcAP1*, and *JcAP3* were significantly up-regulated (Figure 8). *JcSOC1* was also strongly up-regulated in the transgenic *in vitro* flowering lines (Figure 8), indicating that it is a target of *JcFT*, which is consistent with the findings that *SOC1* and *AP1* are activated by the FT–FD complex in *Arabidopsis *[15,16,34].

## Discussion

Chailakhyan [35] coined the term “florigen” to refer to the floral stimulus, but exactly what contributes florigen remains unclear. Evidence indicating that *Arabidopsis* FT protein acts as a long-distance signal to induce flowering was published half a decade ago [12]. Subsequent findings have led to the now widely accepted view that FT protein is the mobile flowering signal (florigen), or at the very least, a component of it [14]. In the present study, we found that *JcFT* encoded an *FT* homolog in *Jatropha*, and thus represented a potential flowering activator.

*FT*-like genes have been isolated from many plants. There are two members of the *FT*-like subclade in *Arabidopsis *[36], five in Lombardy poplar [37], ten in soybean [38], three in chrysanthemum [39], thirteen in rice, and fifteen in maize [40]. In *Jatropha*, we cloned only one member of the *FT*-like subclade, and only one *FT*-like gene was identified in the whole genome sequence data of *Jatropha *[41,42]. Many transgenic plants overexpressing *FT* homologs exhibit an early flowering phenotype [22,38,39,43,44], suggesting a conserved function of *FT* homologs in flowering induction in different plant species.

Although the leaf is generally expected to be the site where a florigen gene is translated into protein [13], many *FT*-like genes are abundantly expressed in reproductive organs, such as flowers and immature siliques in *Arabidopsis *[28], flowers and pods in soybean [38], capsules in poplar [37], inflorescence axes in *Curcuma kwangsiensis *[32], and flowers and berries in grapevine [45]. In the present study, *JcFT* was mainly expressed in flowers, fruits, and seeds, with the highest expression level in female flowers, suggesting that *JcFT* may be involved in the development of reproductive organs. In fact, *FT*-like genes in various species play multifaceted roles in plant development in addition to the crucial role of *FT* homologs in flowering induction [10].

Transgenic *Arabidopsis* ectopically expressing the *JcFT* exhibited an early flowering phenotype compared with the control plants (Figures 4 and 5). Similarly, transgenic *Jatropha* overexpressing *JcFT* flowered *in vitro* during regeneration (Figure 6), which may have resulted from the up-regulation of flowering gens downstream of *JcFT* (Figure 8). Unexpectedly, the transgenic *Jatropha* flower buds that were produced *in vitro* failed to develop normally into mature flowers. Many flower buds were abortive, and only a few developed into abnormal flowers. We supposed that the floral abnormalities and the failure of regeneration of the *35S::JcFT* transgenic *Jatropha* plants could resulted from the ectopic overexpression of *JcFT* driven by the strong constitutive 35S promoter. Consistent with this hypothesis, by using a phloem-specific promoter SUC2, we successfully obtained SUC2::*JcFT* transgenic *Jatropha* shoots, which were grafted onto rootstocks of wild-type *Jatropha* seedlings. The grafted SUC2::*JcFT* transgenic *Jatropha* plants flowered earlier than did wild-type plants, and produced normal flowers (Additional file 2: Figure S2). Therefore, the production of normal transgenic *Jatropha* overexpressing *JcFT* for use in molecular breeding programs of *Jatropha* will likely require the use of weaker constitutive promoters [43], tissue-specific promoters [46], or inducible promoters [47] to confine the expression of the transgene *JcFT* to shoot meristems at an appropriate level. In addition, a loss of function analysis with a RNA interference construct targeted at *JcFT* will be necessary to determine the exact function of *JcFT* in *Jatropha* flowering.

## Conclusions

The *FT* homolog of the biofuel plant *Jatropha* was isolated and characterized in the present study. *JcFT* is mainly expressed in the reproductive organs, including female flowers, fruits, and seeds. *JcFT* also induced early flowering in transgenic *Arabidopsis* and *Jatropha,* indicating that *JcFT* acts as a flowering promoter in *Jatropha*.

## Materials and methods

### Plant materials and growth conditions

The roots, stems, young leaves, mature leaves, flower buds, flowers, and fruits of *Jatropha curcas* L. were collected during the summer from the Xishuangbanna Tropical Botanical Garden of the Chinese Academy of Sciences, Mengla County, Yunnan Province in southwestern, China. Mature seeds were collected in autumn. All tissues prepared for qRT-PCR were immediately frozen in liquid N_2_ and stored at -80°C until use.

WT *Arabidopsis thaliana* ecotype Columbia (Col-0), the *ft-10* mutant (a gift from Dr. Tao Huang, Xiamen University), and the transgenic lines were grown in peat soil in plant growth chambers at 22 ± 2°C under a 16/8 h (light/dark) or 8/16 h (light/dark) photoperiod, with cool-white fluorescent lamps used for lighting. Transgenic plants in the T_2_ homozygous generation were selected to examine flowering time and other phenotypes. For each genotype, ten plants were used to for characterization: the number of leaves was counted along with the number of days between sowing and when the first flower bud was visible.

### Cloning of *JcFT* cDNA

Total RNA was extracted from the leaves of flowering *Jatropha* using the protocol described by Ding *et al*. [48] First-strand cDNA was synthesized using M-MLV-reverse transcriptase from TAKARA (Dalian, China) according to the manufacturer’s instructions. To clone the conserved region of *JcFT* cDNA, a pair of primers, ZF632 and ZF633, was designed according to the conserved regions of *FT* homologs from other plant species using the Primer Premier 5 software. The PCR products were isolated, cloned into the pMD19-T simple vector (TAKARA, Dalian, China), and sequenced. The cloned sequence was used to design gene-specific primers (GSPs) to amplify the cDNA 5′ and 3′ end. The primers were listed in Table S1. First round PCR and nested amplification were performed according to the instructions provided in the SMART™ RACE cDNA Amplification Kit User Manual (Clontech). The PCR products were subsequently cloned into pMD19-T and sequenced.

The full length *JcFT* cDNA was obtained by PCR using the primers JcFT-F and JcFT-R, which introduced *Kpn*I and *Sal*I recognition sites, respectively, to facilitate the transformation of *JcFT* into *Arabidopsis* and *Jatropha*. The PCR products were subsequently cloned into the pMD19-T and sequenced.

### Sequence and phylogenetic analyses

Sequence chromatograms were examined and edited using Chromas Version 2.23. Related sequences were identified using BLAST (http://www.ncbi.nlm.nih.gov/BLAST/). To determine the amino acid identities, sequences from the alignment were pairwise compared using DNAMAN 6.0. A phylogenetic tree based on the protein sequences was constructed using MEGA5.0 (http://www.megasoftware.net). The amino acid sequences of the PEBP family were assembled using ClustalX. A Neighbor–Joining phylogenetic tree was generated with MEGA 5.0 using the Poisson model with gamma-distributed rates and 10000 bootstrap replicates. The molecular weight and isoelectric point of the protein were analyzed on-line using ExPASy (http://web.expasy.org/compute_pi/).

### Plant expression vector construction and *Arabidopsis* and *Jatropha* transformation

To construct the plant overexpression vector 35S::*JcFT*, the *JcFT* sequence was excised from the pMD19-T simple vector using the restriction enzymes *Kpn*I and *Sal*I and then cloned into the pOCA30 vector containing the CaMV 35S promoter and the 35S enhancer. The SUC2 promoter was obtained by PCR from *Arabidopsis* genomic DNA using primers SUC2-F and SUC2-R, which introduced *Hind*III and *Kpn*I recognition sites, respectively. The PCR products were cloned into pMD19-T and sequenced. To construct the SUC2::*JcFT* plasmid, the 35S promoter of the vector containing 35S::*JcFT* was placed with the SUC2 promoter using the restriction enzymes *Hind*III and *Kpn*I. The fidelity of the construct was confirmed by PCR and restriction digestion.

Transformation of WT Col-0 and *ft-10* mutant plants with *Agrobacterium* strain EHA105 carrying the recombinant constructs was performed using the floral dip method [49]. Transgenic seedlings were selected for kanamycin resistance and confirmed by genomic PCR and RT-PCR.

Transformation of *Jatropha* with *Agrobacterium* strain LBA4404 carrying the overexpression construct was performed according to the protocol described by Pan *et al. *[33].

### Expression analysis by qRT-PCR

*Jatropha* total RNA was extracted from frozen tissue as described by Ding *et al. *[48] *Arabidopsis* total RNA was extracted from frozen tissue using TRIzol reagent (Transgene, China). First-strand cDNA was synthesized using the PrimeScript® RT Reagent Kit with gDNA Eraser (TAKARA, Dalian, China) according to the manufacturer’s instructions. qRT-PCR was performed using SYBR® Premix Ex Taq™ II (TAKARA) on a Roche 480 Real-Time PCR Detection System (Roche Diagnostics).

The primes used for qRT-PCR are listed in Table S1. qRT-PCR was performed using two independent biological replicates and three technical replicates for each sample. Data were analyzed using the 2^–ΔΔCT^ method as described by Livak and Schmittgen [50]. The transcript levels of specific genes were normalized using *Jatropha Actin1* or *Arabidopsis Actin2*.

### Availability of supporting data

All the supporting data of this article are included as additional files (Additional file 1: Figure S1; Additional file 2: Figure S2; Additional file 3: Table S1).

## Abbreviations

AP1: APETALA1; FT: FLOWERING LOCUS T; LFY: LEAFY; LD: long day; SD: short day; SOC1: SUPPRESSOR OF OVEREXPRESSION OF CONSTANS 1; SUC2: sucrose transporter 2; qRT-PCR: quantitative reverse transcriptase-polymerase chain reaction.

## Competing interests

The authors declare that they have no competing interests.

## Authors’ contributions

CL and ZFX conceived the experiment and drafted the manuscript. LL cloned *JcFT* cDNA. CL constructed the vector and performed *JcFT* expression pattern analysis, *Arabidopsis* and *Jatropha* transformation, the transgenic plants bioassays. QF contributed to the data processing. LN collected the various *Jatropha* tissue samples. All authors read and approved the final manuscript.

## Authors’ information

CL and LN are PhD students, LL was a master student at the time of study, and QF is an associate professor, and ZFX is a professor and head of the laboratory.

## Supplementary Material

Additional file 1: Figure S1 Semi-quantitative **(A)** and quantitative **(B)** RT-PCR analysis of flowering genes downstream of *FT* in WT and transgenic *Arabidopsis. Arabidopsis* seedlings were collected 20 days after germination. For semi-quantitative RT-PCR, 25 cycles were used for the reference gene *AtActin2*, and 30 cycles were used for the target genes. The qRT-PCR results were obtained from three technical replicates for each sample. Values were normalized using *AtActin2* gene as a reference. The mRNA level in WT was set as the standard with a value of 1. Click here for file

Additional file 2: Figure S2 Early flowering of SUC2::*JcFT* transgenic *Jatropha*. Transgenic shoot grafted onto a non-transgenic rootstock showing the precocious flowers (red oval) forty days after grafting. Red arrows indicate the graft sites. Click here for file

Additional file 3: Table S1 Primers used in this study. Click here for file
