# Supplementary figures and images for: Isolation and functional characterization of JcFT, a FLOWERING LOCUS T (FT) homologous gene from the biofuel plant Jatropha curcas
Source: BMC Plant Biol. 2014 May 8;14:125. doi: 10.1186/1471-2229-14-125 (PMC4036407; doi:10.1186/1471-2229-14-125)

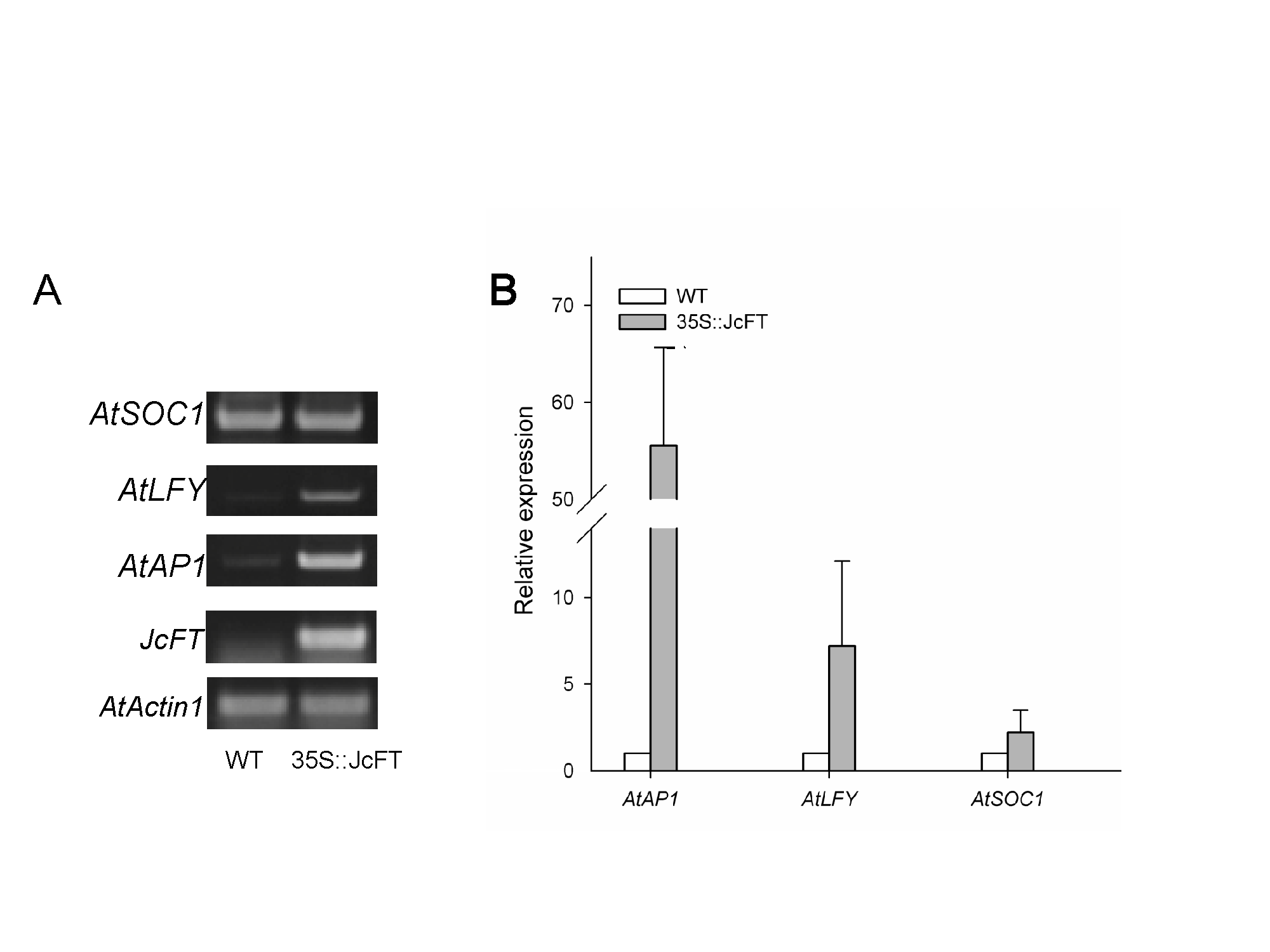

Supplement: Additional file 1: Figure S1 — Semi-quantitative (A) and quantitative (B) RT-PCR analysis of flowering genes downstream of FT in WT and transgenic Arabidopsis. Arabidopsis seedlings were collected 20 days after germination. For semi-quantitative RT-PCR, 25 cycles were used for the reference gene AtActin2, and 30 cycles were used for the target genes. The qRT-PCR results were obtained from three technical replicates for each sample. Values were normalized using AtActin2 gene as a reference. The mRNA level in WT was set as the standard with a value of 1. [file 1471-2229-14-125-S1.tiff]

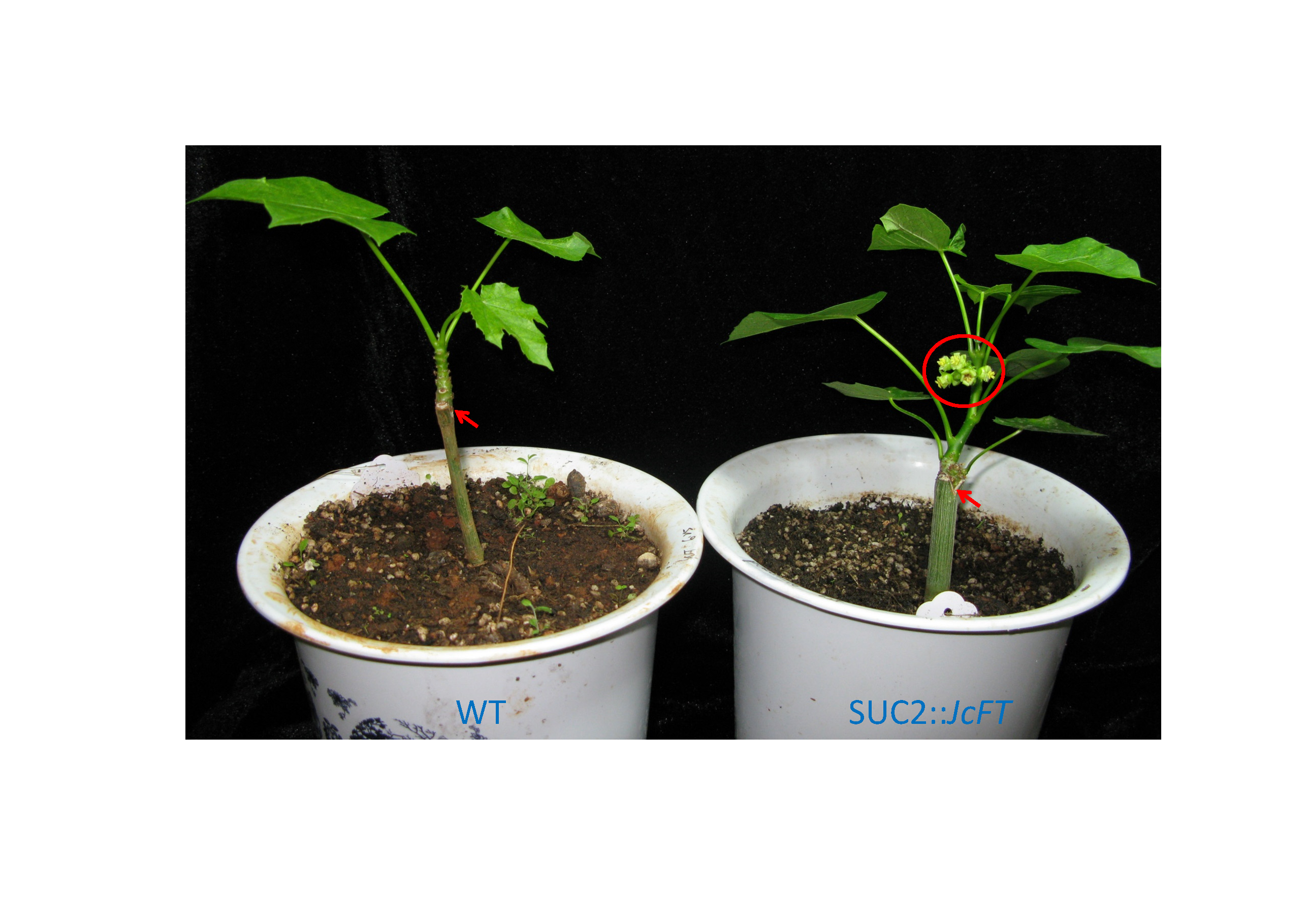

Supplement: Additional file 2: Figure S2 — Early flowering of SUC2::JcFT transgenic Jatropha. Transgenic shoot grafted onto a non-transgenic rootstock showing the precocious flowers (red oval) forty days after grafting. Red arrows indicate the graft sites. [file 1471-2229-14-125-S2.tiff]
