# Supplementary material for: Isolation and functional characterization of JcFT, a FLOWERING LOCUS T (FT) homologous gene from the biofuel plant Jatropha curcas
Source: BMC Plant Biol. 2014 May 8;14:125. doi: 10.1186/1471-2229-14-125 (PMC4036407; doi:10.1186/1471-2229-14-125)
Supplement: Additional file 3: Table S1 — Primers used in this study. [file 1471-2229-14-125-S3.docx]

**Supplementary Table S1 Primers used in this study.**

| Name | Sequences (from 5’ to 3’) |
| --- | --- |
| ZF632 | TGC ATT GGT TGG TGA CTG ATA T |
| ZF633 | CTT CCD CCG GAG CCA CTC TCC CTC TG |
| *JcFT* 5’ RACE GSP1 | AGC TCA CAG CCA TTG TTA ACC TCT CT |
| *JcFT* 5’ RACE GSP2 | GGG TCT AAA ACA TCC CCA ATC ACA CG |
| *JcFT* 3’ RACE GSP | ACC AGG GTG GCG TCA GAA TTT CAA CA |
| JcFT-F | GCG GTA CCG TAA TGC CTA GGG ATC AA |
| JcFT- R | AAA GTC GAC TCA CCG TCT CCG TCC TCC G |
| SUC2-F | GAT AAG CTT CAT GCA AAA TAG CAC ACC AT |
| SUC2-R | ACG GTA CCA TTT GAC AAA CCA AGA AAG TAA G |
| *JcFT* qRT-PCR F | AGG CAG ACC GTG TAT CCA CCA G |
| *JcFT* qRT-PCR R | ACT GAA TCA CCG TCT CCG TCC TC |
| *JcLFY* qRT-PCR F | GGA TAA GAT ACT ACA CAG CAG CGA |
| *JcLFY* qRT-PCR R | TAA CCC TTC TTG AGA GAG AGC ATC |
| *JcAP1* qRT-PCR F | GGG TTA TTT TGA GGA AAG AAG AGG A |
| *JcAP1* qRT-PCR R | AAA CAA TCA AAG CAA CCT CAG CAT C |
| *JcSOC1* qRT-PCR F | TTC TTG GAC GGC AAC GCT TA |
| *JcSOC1* qRT-PCR R | CTC TCG GAA AAG TGT GGG ATC |
| *JcAP3* qRT-PCR F | TCT CTT CGG TTT TGT AGT AGT GGG TTT |
| *JcAP3* qRT-PCR R | AGA ACA GTG AGT TGC TTG AGC TTT TTT |
| *JcActin1* qRT-PCR F | CTC CTC TCA ACC CCA AAG CCA A |
| *JcActin1* qRT-PCR R | CAC CAG AAT CCA GCA CGA TAC CA |
| *AtLFY* qRT-PCR F | TCT CTC CCA AGA AGG GTT AT |
| *AtLFY* qRT-PCR R | GTA GTG TCG CAT TTT AGG CT |
| *AtAP1* qRT-PCR F | GAC GTC AAT ACA AAC TGG TCG A |
| *AtAP1* qRT-PCR R | GGA GAT GGC TGA TGA GAG AGC |
| *AtSOC1* qRT-PCR F | CTA AAC GTA AAC TCT TGG GA |
| *AtSOC1* qRT-PCR R | CAG AAC TTG GGC TAC TCT CT |
| *AtActin2* qRT-PCR F | TGT GCC AAT CTA CGA GGG TTT |
| *AtActin2* qRT-PCR R | TTT CCC GCT CTG CTG TTG T |
